# Fibre-Reinforced Geopolymer Concretes for Sensible Heat Thermal Energy Storage: Simulations and Environmental Impact

**DOI:** 10.3390/ma14020414

**Published:** 2021-01-15

**Authors:** Domenico Frattini, Alessio Occhicone, Claudio Ferone, Raffaele Cioffi

**Affiliations:** 1 Graduate School of Energy and Environment, Seoul National University of Science and Technology, Gongneung-ro 232, Nowon-gu, Seoul 01811, Korea; 2 Department of Engineering, University Parthenope of Naples, Centro Direzionale di Napoli Is. C4, 80143 Napoli, Italy; alessio.occhicone@uniparthenope.it (A.O.); raffaele.cioffi@uniparthenope.it (R.C.)

**Keywords:** AHP, carbon fibres, conductivity, geopolymers, LCA, sustainability, thermal storage

## Abstract

Power plants based on solar energy are spreading to accomplish the incoming green energy transition. Besides, affordable high-temperature sensible heat thermal energy storage (SHTES) is required. In this work, the temperature distribution and thermal performance of novel solid media for SHTES are investigated by finite element method (FEM) modelling. A geopolymer, with/without fibre reinforcement, is simulated during a transient charging/discharging cycle. A life cycle assessment (LCA) analysis is also carried out to investigate the environmental impact and sustainability of the proposed materials, analysing the embodied energy, the transport, and the production process. A Multi-Criteria Decision Making (MCDM) with the Analytical Hierarchy Process (AHP) approach, taking into account thermal/environmental performance, is used to select the most suitable material. The results show that the localized reinforcement with fibres increases thermal storage performance, depending on the type of fibre, creating curvatures in the temperature profile and accelerating the charge/discharge. High-strength, high-conductivity carbon fibres performed well, and the simulation approach can be applied to any fibre arrangement/material. On the contrary, the benefit of the fibres is not straightforward according to the three different scenarios developed for the LCA and MCDM analyses, due to the high impact of the fibre production processes. More investigations are needed to balance and optimize the coupling of the fibre material and the solid medium to obtain high thermal performance and low impacts.

## 1. Introduction

In recent decades, Solar Thermal Power Plants were developed at a large scale to indirectly convert concentrated solar energy into green electricity [1]. These plants work at high temperatures, and as frequently occurs for renewable energy sources, they are highly dependent on the availability of solar radiation, i.e., only during the day [2]. Hence, a reliable and efficient thermal storage system is mandatory to extend the productivity of these energy plants. Sensible heat thermal energy storage (SHTES) is applied for a high-temperature range, i.e., >573 K. In particular, the most feasible and economic storage materials for SHTES are represented by high performance concretes [3]. However, in the long term, they suffer from thermal fracture and failure due to thermal stress and non-uniform temperature distribution during charging/discharging steps. To solve these issues, high-strength-high-conductivity long fibres can be added to increase heat diffusion and mechanical strength. Recently, geopolymer concretes are reported to have a higher thermal stress resistance in repeated heating/cooling cycles at high temperatures [4,5,6], but improved thermal properties are required to compete with reference benchmarks [7]. Many numerical and simulation approaches have been used in literature to predict, evaluate, and enhance their performance [8,9,10]. Among them, FEM is the most useful to obtain a reliable image of spatial distribution inside storage material because this is of fundamental importance for module design and performance analysis [11,12]. Fibre spacing, pattern, and thermal properties are the main drivers for SHTES concrete reinforcement and fibre embedding; hence, an efficient simulation of the temperature distribution during the charge/discharge cycle of a thermal storage unit based on fibre-reinforced concretes is necessary. Moreover, considering large scale plants, integrated storage systems, and desired lifetime, a comprehensive design must take into consideration not only the thermal properties of the solid storage medium and its simulated performances but also the environmental impact and sustainability of the materials and processes involved in its production.

Geopolymers are innovative binders that have been extensively studied in recent years consisting of amorphous to semi-crystalline aluminosilicates synthesized using alkaline solutions and solid precursors such as low-Ca fly ash [13], calcined clays [14,15,16], and other industrial, and natural waste [17,18,19,20].

These materials show excellent mechanical properties, low shrinkage (low-Ca precursors), thermal stability, freeze-thaw, acid and fire resistance, long term durability, and recyclability [21,22]; thus, they are a potential alternative to traditional Portland cement in selected applications because in many cases they have also a reduced environmental impact.

On the other hand, owing to their ceramic nature, they have relatively low toughness and low flexural strength and, to improve these properties, geopolymer matrix composite materials have been prepared and studied. Plenty of studies [23,24] have been produced on this topic, and many types of fillers have been tested, such as particulate and various kinds of short and continuous fibres. Novel geopolymer matrix composites and hybrids have also been obtained by the in situ co-reticulation of a geopolymer matrix with an epoxy-based organic resin [25,26,27,28,29,30].

In addition to the mechanical performance, geopolymer matrix composites can be used to modify geopolymer thermal properties, such as thermal conductivity and fireproofing [31], e.g., sustainable geopolymer concrete with good thermal insulation properties were obtained by incorporating recycled expanded polystyrene spheres in a metakaolin based geopolymer matrix, together with a waste-derived filler [32]. Considering this background, it is demonstrated that geopolymer concretes represent a high potential alternative material for SHTES based on solid media due to their versatile properties, formulations, and feasibility.

In this work, a geopolymer concrete is modelled as a solid medium for SHTES. The enhancement of thermal properties and temperature distribution are obtained by adding fibres with high conductivity in an ordered arrangement around the heat exchanger pipe. Temperature contours, time evolution, and thermal performance of geopolymer concretes are compared with and without fibres to demonstrate the effect of fibres and fibre materials.

In addition to the simulation of the behaviour of the new geopolymer materials, for their use in thermal energy storage, and the comparison with previous literature, an analysis of the environmental impact that the production of 1 m^3^ of material has on the ecosystem was performed. This type of approach was useful to understand the technical and environmental limits of the new materials designed for thermal storage units. The environmental impact assessment was carried out following the rules imposed by the life cycle assessment (LCA), following a cradle-to-gate approach under the conditions valid for the assessment of the Environmental Product Declaration (EPD^®^) for possible future industrialization in Italy.

The International EPD^®^ System is a global program that communicates verified, transparent, and comparable information about the life cycle environmental impact of products. EPD is based on ISO 14025 (ISO 14025:2006 Environmental labels and declarations—Type III environmental declarations—Principles and procedures) and EN 15804 (EN 15804: 2012 Sustainability of construction works, Environmental product declarations, Core rules for the product category of construction products) standards. For this reason, EPD is among the most accepted methods that return, in a standardized and comparable way, the results of the LCA related to a product, a process or an activity [33].

To give support in the decision-making process at the industrial level, the thermal and environmental performances obtained were also evaluated through the Super-Decisions simulation program. This software provides a powerful methodology for combining judgment and data to effectively rank options and predict outcomes. Several authors [34,35] showed that, following the combined LCA-Analytical Hierarchy Process (AHP) Multi-Criteria Decision Making (MCDM) approach, it is possible to obtain the best evaluation of the behaviour of materials from the point of view of performance and environment. This kind of approach has been used by the scientific community, due to its simplicity and robustness for sustainable evaluation [36] and has already been used to choose sustainable materials based on their features [37].

## 2. Materials and Methods

### 2.1. Model Design of the Fibre-Reinforced SHTES Unit and Simulation Conditions

The storage module is a complex parallelepiped with an embedded heat exchanger composed by tubes in square arrangement [38]. This structure is often decomposed as a repetition of differential storage elements due to module symmetry. The differential storage element for calculations is usually a hollow cylinder with a cross-section area equivalents to that of a square [9,38,39]. Differently from the cited references, the geometry is not further reduced to a 1D radial problem because the external square represents a real physical limit and, from the thermal point of view, this could be incorrect as the temperature diffusion (e.g., at the corners) could be uneven in certain directions, and could be undetected during the transient simulation. Moreover, a 1D radial modelled element does not allow the local enhancement by inserting discrete objects to create multi-conduction areas for a thermally engineered design.

**Figure 1:**
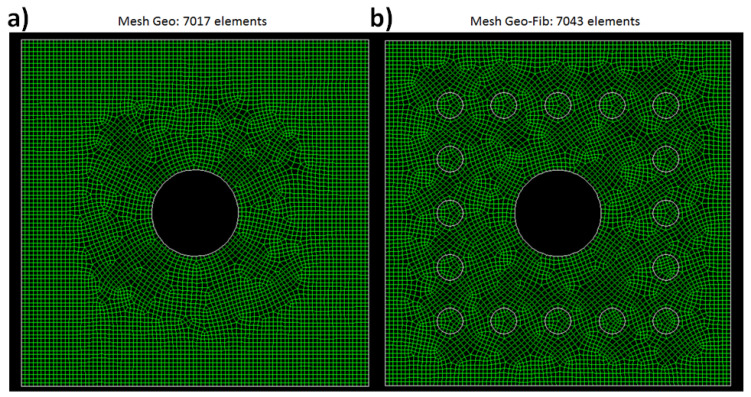
Cross-section mesh of the storage element used for simulations. (**a**) Mesh Geo without fibres. (**b**) Mesh Geo-Fib with fibres.

Therefore, as an extension of previous literature [7,40], in this work, a square-based parallelepiped of unit length (1 m) is the differential storage element for FEM simulations. The geometric mesh was modelled in GAMBIT 2.3.16. The new feature is the arrangement of 16 bunches of fibres in squared pitch around the central heat exchanger pipe as shown in Figure 1.

The storage mesh has an 8 × 8 cm^2^ section area; fibres are centrally spanned on a 5 × 5 cm^2^ square around the tube. The tube diameter is 2 cm, fibre bunch diameter is 0.6 cm, and the interspace between each bunch is 0.65 cm. Further details on the mesh are reported elsewhere [7]. The storage material is a geopolymer concrete (G), while two types of fibres, i.e., Carbon fibre (FibC) or Nickel fibre (FibNi), are considered as reinforcement. Their properties are listed in Table 1. These two types of fibres, i.e., a metal-based and a carbon-based one, were selected because they are two common commercial fibres, not very expensive, and show the same thermal conductivity but different density and specific heat capacity (hence, a different diffusivity). So, it was interesting to compare them for this specific application. Moreover, they are also already used to reinforce concretes and building materials to improve the mechanical resistance and hence the lifetime of the material, aspects that are not directly investigated in this work. Given the improved mechanical resistance and durability, more information about the thermal performance and sustainability of SHTES is needed when considering these fibres.

For FibC and FibNi, the thermal properties were retrieved from [41], while those for G are from [7]. Thermal diffusivity α and volumetric thermal capacity *C_vol_* were simply calculated from *ρ*, *c*, and *k* as follows:

(1) $$\alpha =\frac{k}{\rho \cdot c}$$

(2) $$C_{vol}=\rho \cdot c$$

Sensible heat storage FEM simulations were run using the computational fluid dynamics CFD software ANSYS Fluent (v. 6.3.26, ANSYS Inc., Canonsburg, PA, USA). Assumptions, governing equations, storage cycle, boundary, and initial conditions are the same as those reported in the literature [7,10,40] and were used without relevant modifications. In brief, the maximum temperature difference for storage was 40 K, i.e., from 623 to 663 K, all the materials were considered isotropic, thermal properties were considered constant within the small temperature range, and the unit was considered perfectly insulated thermally. The imposed thermal cycle was 3600 s of charge/discharge and 3600 s of storage (buffering time or “break”). The time step for simulations was 2 s, convergence criteria were set at 10^−4^, and the built-in Monitor function of Fluent was used to take the contour profiles of temperature distribution at 5, 20, 40, and 60 min during charge/discharge with a temperature accuracy (colour scale) of 2 K, while one of the external edges of the module was selected to record the average T profile at the wall.

### 2.2. LCA Analysis

Comparative LCA analysis was carried out on the three geopolymer concretes studied in this work, and on materials studied in previous work for similar applications. In particular, a plain cement concrete (C) [7], a modified concrete with marble sludge (PA0), the same modified concrete with 20% by weight of recycled plastic (PA20) [7], and the material named A4 developed by Guo et al. [42] were compared against the coal fly ash-based geopolymer (G), the same coal fly ash-based geopolymer matrix composite with Carbon fibres (FibC) or with Nickel fibres (FibNi), as described in the previous section. In Table 2, the compositions of all tested materials are reported.

The LCA was performed according to ISO 14040:2006 (ISO 14040:2006 Environmental Management—Life Cycle Assessment—Principles and Framework) with the Simapro© software (v. 8.5.2, PRé Sustainability, Amersfoort, The Netherlands).

In this work, the boundary system involved raw materials, transport, and manufacturing and was based on 1 m^3^ of material production.

In a specific view:

- The LCA was performed in a way that considered the contribution of the raw materials for all the different mixtures; the production processes have some energy consumption in common, in particular material milling, mixing, and element cutting are similar for each material, so they have the same value for all the products. The difference in the process is the curing necessary for geopolymer products, which need 24 h in a climatic chamber at 60 °C to complete reticulation reaction and hardening. In this case, the electricity consumption linked to this step was estimated using the Italian energetic mix as in [43].
- The impacts of the raw materials were estimated including extraction and all the necessary processes preliminary to their use. For fly ash, just the transportation contributed to the impacts, because it can be used directly in the production process and can be considered as a no-impact material, as demonstrated in previous works [43,44]. In the case of recycled plastic, just the milling and the transport were estimated, with a negative global contribution for different environmental impact items such as global warming potential (all the contributions are shown in Appendix A, Table A1, Table A2, Table A3, Table A4, Table A5, Table A6 and Table A7). This is because the environmental impacts due to traditional management of plastic (disposal mix: in landfills, recycling, composting, burning, etc.) are avoided. For the calcium aluminate cement and the superplasticizer, the environmental impacts were calculated based on the EPD^®^ 830 çimsa RESISTO40 following ISO14040/44 [45] and the European Federation of Concrete Admixtures Associations Ltd. (EFCA) EPD^®^ according to ISO14025:2011-10 [46], respectively. The estimated impacts of PAN fibre production were also taken from the literature [47].
- All other material impacts were taken from Simapro Ecoinvent 3 database.
- The transportation stage was considered for the delivery of the raw materials to the plant. In particular, based on the average availability of materials in Europe, an average transport distance up to 100 km for all the starting materials was considered. It is worth noting that this estimation had little effect on the overall production impacts of each system, bordering on undetectable.

No treatment of “the end of life” was considered for any of the products, except for the recycled materials used as precursors. In this case, the missed impacts from landfill disposal were evaluated and inserted with a negative value. So, a cradle-to-gate approach was considered, following the EPD.

SimaPro 8.5.2 © is equipped with different methods for assessing impacts. In particular, the ReCiPe (2016) Midpoint method was chosen in the present work. This is a method for Life cycle impact assessment (LCIA) that translates emissions and resource extractions into a limited number of environmental impact scores utilizing the so-called characterization factors [48]. In this work, it was chosen at the midpoint level to better underline the contribution of all precursors in the environment, without the loss of sensitivity linked to the implementation of all the indicators in the three macro areas that constitute the endpoint level of the method [49].

This LCA method includes 18 midpoint impact categories, but, in this work, the midpoint characterization is shown just for some of the items (the remaining ones are reported in Appendix A):

- Climate change: Global Warming Potential (GWP), which quantifies the integrated infrared radiative forcing increase in greenhouse gas (GHG), expressed in kg CO_2_-eq (IPCC 2013).
- Stratospheric ozone depletion: The ozone-depleting potential (ODP), expressed in kg CFC-11 equivalents, was used as a characterization factor at the midpoint level. ODP refers to a time-integrated decrease in stratospheric ozone concentration over an infinite time horizon [50].
- Particulate matter: Quantification of the impact of premature death or disability that particulates/respiratory inorganics have on the population, in comparison to PM_2.5_. This includes the assessment of primary (PM_10_ and PM_2.5_) and secondary PM (including the creation of secondary PM due to SO_x_, NO_x_, and NH_3_ emissions) and CO [51].
- Photochemical ozone formation: human health ozone formation potential (HOFP) is expressed in kg NO_x−eq_. The change in ambient concentration of ozone after the emission of a precursor (nitrogen oxides (NO_x_) or non-methane volatile organic compounds (NMVOC)) was predicted with the emission–concentration sensitivity matrices for emitted precursors from the global source-receptor model, TM5-FASST [52].
- Terrestrial acidification: For the midpoint characterization factors of acidifying emissions, the fate of a pollutant in the atmosphere and the soil was calculated as in [53]. Acidification potentials (AP) are expressed in kg SO_2_-eq. Changes in acid deposition, following changes in air emission of NO_x_, NH_3_, and SO_2_, were calculated with the GEOS-Chem model [54].
- The midpoint indicator for fossil resource use, determined as the Fossil Fuel Potential (FFP in kg oil-eq), is defined as the ratio between the higher heating value of a fossil resource and the energy content of crude oil [55].

The limitation of these impact factors is for quicker analysis, but all the environmental impact categories are reported in Appendix A.

### 2.3. Super-Decisions AHP Analysis

The six parameters shown in LCA analysis were considered Super-decisions sub-criteria. At the end of environmental analysis, it was possible to investigate the best materials from the point of view of both thermal and environmental performance. To do this, Super-decisions free software (v. 3.2, Creative Decisions Foundation, Pittsburgh, PA, USA) was used. This software provides tools to create and manage AHP and Analytic Network Process (ANP) models, enter judgments, obtain results, and perform sensitivity analysis on the results. In the present work, a hierarchical approach (AHP) was chosen. In this kind of analysis, levels are arranged in descending order of importance. The elements in each level are compared according to dominance or influence for the elements in the level immediately above that level [56].

**Figure 2:**
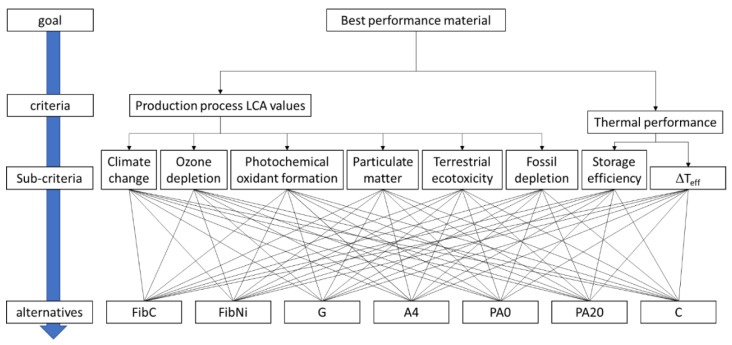
Super-decisions Analytical Hierarchy Process (AHP) model to select the best sensible heat thermal energy storage (SHTES) material.

Figure 2 shows the graph of the hierarchical levels with which the calculations were carried out. Specifically, we have the most suitable material that derives from its thermal performance and from the environmental impact generated by the production of the material itself.

The chosen criteria consider three different scenarios: (i) the material production process LCA values have the same importance of thermal performance; (ii) the LCA values of materials have a weight four times greater than that of thermal performance; (iii) the weight of thermal performance is four times that of LCA values. The first scenario can represent the case of the short lifetime of the plant; on the other hand, the third scenario can represent the case of a very long lifetime, in which the environmental impact of the production process is spread over a long period in the third scenario, and the durability of the materials becomes fundamental, but this is not the subject of the present work, and this factor could likely see fibre-reinforced materials prevail over the unreinforced ones. In the sub-criteria, a different weight was given to LCA parameters and thermal storage variables. The rank of the different variables was chosen according to the authors’ experience [57], based on the LCA results and the thermal performance obtained from the simulations (Appendix A and Appendix B).

The scale used to compare different criteria is that suggested by Saaty 2003 [58]: (1) equal, (2) between equal and moderate, (3) moderate, (4) between moderate and strong, (5) strong, (6) between strong and very strong, (7) very strong, (8) between very strong and extreme, (9) extreme. In Table 3, the weights of the 8 variables considered in the sub-criteria are reported. Values obtained for each material (alternative) are reported in Appendix B (Table A8, Table A9, Table A10, Table A11, Table A12, Table A13, Table A14, Table A15, Table A16, Table A17, Table A18 and Table A19).

The alternatives should be compared pairwise as well: as a result, a positive reciprocal matrix for the alternatives should be designed. All the judgements over the criteria and alternatives have to be consistent. This means that the inconsistency index for each element of the reciprocal matrices should not exceed 0.1 [59].

## 3. Results and Discussion

### 3.1. Thermal Storage Charge/Discharge Temperature Profiles, Maps and Performance of Fibre-Reinforced Geopolymer Concretes

**Figure 3:**
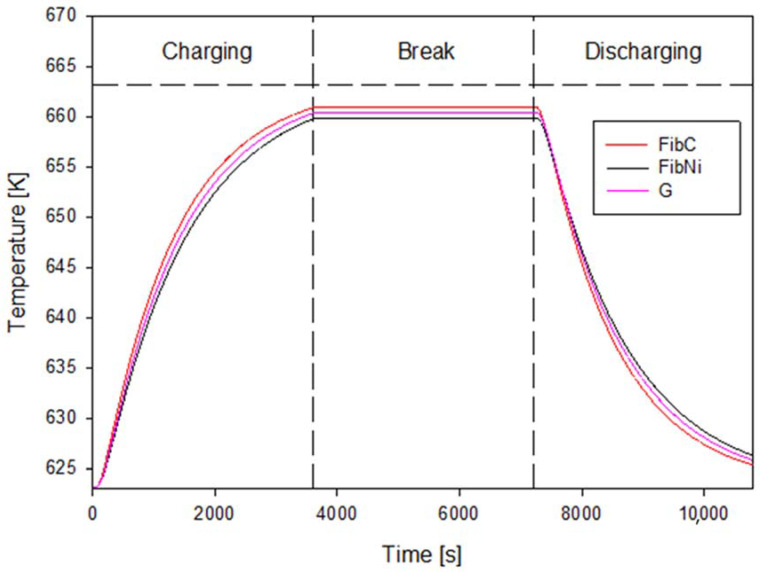
Temperature profile at the wall for the geopolymer concrete with/without fibres.

In Figure 3, the average temperature profile at the wall during a thermal cycle is shown for the three different storage elements investigated. As noticeable, in all cases, the elements could charge and discharge very fast. In detail, the element reinforced with carbon fibres, FibC, attained the highest temperature after the charging step and the deepest discharge state for the given time, i.e., 3600 s for charge/discharge.

However, the thermal behaviour of the three elements was slightly different as the best was FibC, due to the presence of the high conductive carbon fibres, but the worst was FibNi and not the plain geopolymer, G. This would mean that the use of Nickel fibres does not improve thermal performance, although is a metal. In reality, the enhancement of inserting the Nickel fibres relies more heavily on the internal temperature distribution and hence the overall storage efficiency because, as discussed later, due to the high heat capacity of these fibres, the external wall temperature is slightly lower than G, but the distribution of the temperature is completely different. These qualitative conclusions are confirmed by quantitative calculations of thermal performance as reported in Table 4.

The FibC storage element has improved storage efficiency and power density due to the higher effective temperature increase (ΔT_eff_) achieved during charge.

**Figure 4:**
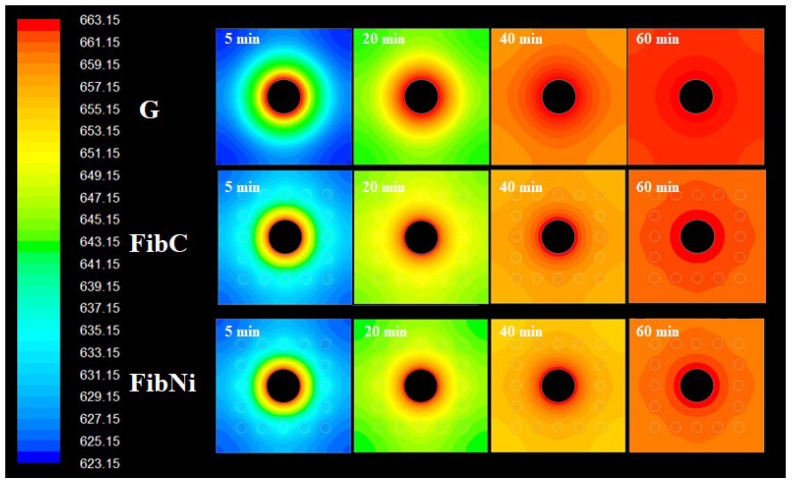
Temperature contours at different charging times for storage elements.

For the local temperature distribution inside the storage element, in Figure 4, the temperature contours at different charging times are compared.

As a consequence of fibre addition, the profile shapes were different. In the plain geopolymer G at the beginning, the temperature contour evolved with a series of concentric waves but, once the wave reached the external wall at the perpendicular direction, the curvature changed and waves slowly propagated to cover corners. At the end of the charge, from the contours, it is evident that temperature distribution was very good but slightly uneven and that the slower propagation at corners was the reason behind this. Differently, when fibres were inserted around the tube, heat propagation was enhanced, and temperature distribution was more uniform at walls as waves proceed smoothly also in corner directions. The two storage elements with fibres both have two well-determined regions, one with uniform temperature all around the walls, and another inner region around the fibres that distribute the heat evenly from the central pipe (that has its heat transfer hot “corona” where the main heat transfer phenomena from the heat transfer fluid take place). The presence of regions with different thermal diffusivity, i.e., fibres and matrix, modified the contour shape and increased the speed of heat propagation near fibres. The lower performances of FibNi, thermal conductivity being the same as for FibC, is ascribed not only to the lower thermal diffusivity but also to the larger volumetric thermal capacity, *C_vol_*, i.e., the quantity of thermal energy required to raise the temperature of 1 m^3^ of storage material by 1 K. This is because the coupling of the matrix G with the carbon fibres seems quite optimal. After all, they have a similar thermal capacity (i.e., same energy required to charge), but the fibres have far higher conductivity and diffusivity (i.e., faster charge); hence, FibC combines the benefits of both.

**Figure 5:**
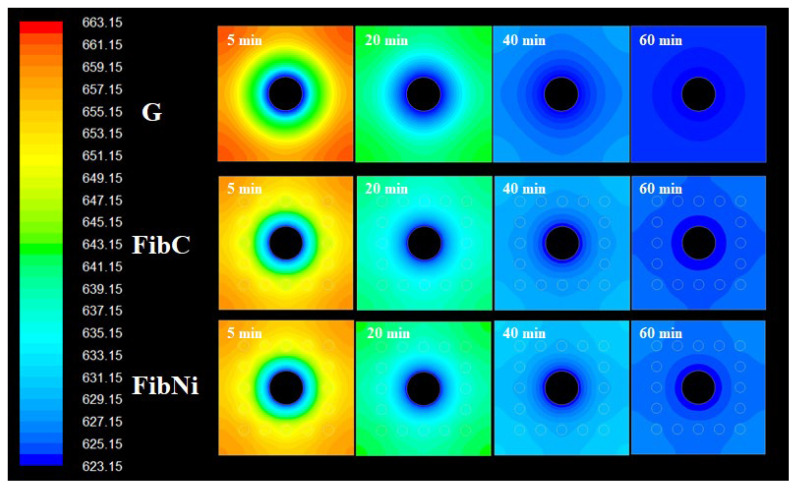
Temperature contours at different discharging times for storage elements.

The discharging behaviour of the three modules was also simulated, and the contours are reported in the following Figure 5.

Discharging behaviour also showed interesting features as it is different for the three materials due to the insertion of the fibres. In G, the discharge was uniform and quite fast, leaving a uniform temperature region all around the walls and another inside the materials. Conversely, the storage elements with fibres both showed a non-circular propagation of the temperature profile in the section due to the fibres. In fact, after discharge, the corners of the elements remained a slightly (i.e., 1–2 K) warmer than the internal region (central pipe corona excluded because it is the “source” of the discharge, similar for all the material). This is probably because the fibres tend to “protect” the element by preventing heat loss, enhancing thermal diffusion because they have their own thermal capacity, i.e., they can store some heat, different from the geopolymer matrix.

However, it should be noted that, although the discharge contours were very different, the average temperature at the wall after discharge (Figure 5) was almost similar for G and FibC, while the FibNi discharged and charged slowly. In fact, at the beginning of the thermal cycle, the three storage modules had the same identical status at the charge, but at the discharge phase, the initial condition of the material was that at the ending of the charging phase; hence, the initial temperature distribution is not the same for all three materials, thus better reflecting the actual behaviour of the modules for real applications. In ideal conditions, the charge and discharge are perfectly reversible; hence, the temperature profiles, e.g., Figure 5, tend to be specular and symmetric, and it is only possible to modulate and tune different times and durations of the charge/discharge phase.

The beneficial effect on thermal performance could be increased by changing the volume loading of the fibres, but an optimal compromise with environmental issues must be carefully considered as it is crucial for the operational life and economic balance of the plant/application, as discussed below.

### 3.2. LCA Environmental Analysis of the Fibre-Reinforced Geopolymer Concretes for SHTES Units Compared with Other SHTES Unit Materials

**Figure 6:**
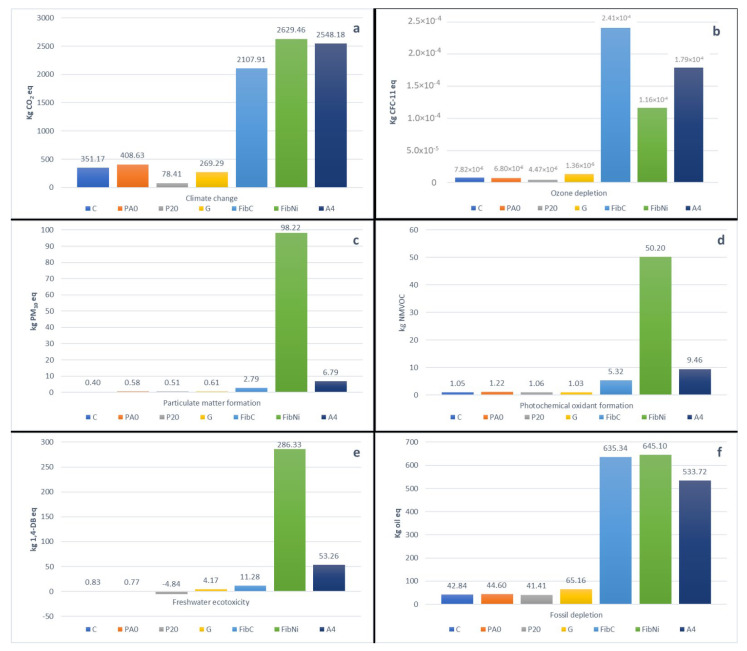
Comparison of environmental impact contribution for the proposed SHTES materials: (**a**) climate change (CO_2_ eq. emission); (**b**) ozone depletion (CFC_11_ eq. emission); (**c**) particulate matter (PM_2.5_ emission); (**d**) photochemical oxidant formation (NMVOC eq. formation); (**e**) terrestrial acidification (SO_2_ eq. production); (**f**) fossil fuel depletion (oil eq. consumption).

The ReCiPe hierarchic midpoint method allowed us to estimate all the impact factors for each material of all the products and the contribution of transport and energy depletion during the production process. The most important impact categories are shown in Figure 6.

As can be observed, the best performances from the environmental point of view were obtained by recycled plastic concrete (PA20), which shows, in some items, negative values for Freshwater ecotoxicity (all the values shown in Appendix A). In this case, both the limestone sludge and the plastic shavings, used in the concrete mix instead of usual disposal, showed a significant reduction in environmental impacts, making them preferable as a green opportunity.

Good and similar impact values were obtained by the concretes based on Portland cement (C) and concrete with the addition of limestone sludge (PA0). Geopolymer shows better performances from the point of view of global warming potential and acidification, and slightly worse values for fossil and ozone depletion, as detected in other geopolymers/Portland concrete comparisons [33,43].

The worst results from an environmental point of view were obtained by the fibre reinforced geopolymers. These types of materials show very good thermal storage capacity, and thanks to the fibres, they could also acquire excellent mechanical performances. In this case, the environmental impact is considerably increased by the fibre production process, which turns out to be quite energy-intensive due to the high temperatures necessary for melting and extrusion. The use of a small aliquot of fibre (4.9% by volume, adopted in thermal simulation) shows increased environmental impact values even 10 times greater than those obtained with pure geopolymer. In this case, the nature of the fibre has a decisive influence on the environmental impact of the entire product. Results comparable to those of fibrous geopolymer composites are obtained by assessing the impacts associated with the use of A4 concrete, although in this case the environmental impact is slightly lower. This type of material turns out to be quite different from ordinary concretes as it is mainly made up of bauxite and basalt and contains a certain amount of graphite and steel fibre, although in lower weight percentage, compared to those elaborated in the present work.

A4 and composite materials exploit virgin raw materials that have a high energy production cost and, therefore, limit their use if compared to the environmental purposes of this work. However, such solutions should not be discarded based on this analysis because their thermal, mechanical, and volume/weight performances are very interesting, and therefore, an assessment over the entire life cycle would be necessary, for example, with an estimation of the lifetime (which is usually considered greater for geopolymers if compared to cement concretes) and with the possible recovery of raw materials at the end of the lifetime.

**Figure 7:**
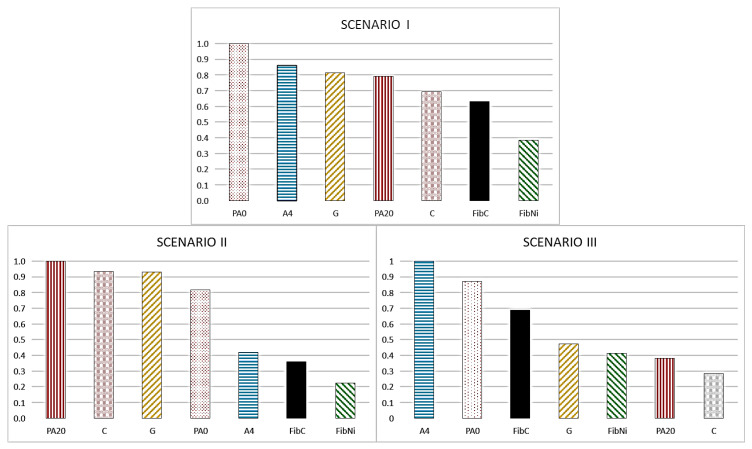
Results of the AHP analysis applied to the proposed SHTES materials, according to the 3 proposed scenarios.

To better understand whether these materials can have a real use from an environmental point of view, it was deemed necessary to carry out a weighted comparison of the technical characteristics of the materials and the environmental impact associated with their production process, by using the Super-decisions software. The Super-decisions multi-criteria analysis results are reported in Figure 7. The values are normalized to the best material for each scenario so that the best option has a value of 1 and the others show a lower level of performance proportional to the best material.

In the first scenario, the best performance was obtained by PA0. This material showed good performance due to its elevated thermal properties and relative intermediate values from an LCA point of view. A4, Geopolymer, and PA20 showed global performance very similar to each other, although their characteristics are very different. A4 is the best thermal material among those studied, but its production is not sustainable. The good performance of Geopolymer is related to its intermediate thermal behaviour coupled with a generally low environmental impact, due to the generally low CO_2_ emission and the reuse of Fly ash waste material. PA20 instead showed the worst thermal behaviour, but a very low environmental impact due to the recycling of plastics and the use of inert sludge derived from marble processing. In this case, plain concrete C also performed slightly better than FibC, characterized by better thermal performance but worse environmental impacts in the production process. Nickel fibre-reinforced geopolymer material showed the worst global performance.

In the second scenario (in which the environmental production impact has the highest importance), the best performance was provided by PA20 concrete, which was better than all the other materials, especially fibre-reinforced ones. In this case, concrete C and Geopolymer had similar performance, better than other materials, starting from PA0 concrete to FibNi material.

In the third scenario, the best material was A4, characterized by the best performances from the storage efficiency and ΔT_eff_ point of view. The performance of PA0 was higher than that of FibC, due to its lower environmental impact and a good value in ΔT_eff_, although the two materials showed similar storage efficiency. Additionally, in this evaluation, Geopolymer showed good performance, better than FibNi and PA20, with the plain concrete C showing the worst. This underlines that, if the industrial investment has a very small period of use, plain concrete could be a good choice due to its low cost (not analysed in this paper), but it is strongly not recommended for long-term entrepreneurial activities.

It is worth noting that these three different scenarios are the extreme conditions. They were evaluated to simulate the decision-making process of the designers when deciding on thermal storage system materials without any sort of “flexibility” and “sensibility” represented by human factors, and the classification exposed above is a rough list based only on mathematical considerations. For example, since solid data on the durability of singular materials are not available, the weight of the environmental impact obtained during the production phase can have a more or less marked relevance depending on material lifetime, but, in any case, the three scenarios can likely represent a good simulation of the possible real cases.

## 4. Conclusions

Among the distinguishing features of this work is the use of a square parallelepiped as a differential storage element instead of a hollow cylinder for a more accurate simulation of heat propagation and temperature distribution inside the SHTES media. This approach allows the exact design of the volume of the fibre-reinforced concretes with enhanced thermal properties for performance evaluation. In fact, in this work, the performances of a geopolymer concrete were estimated with and without high-conductive fibres. The results demonstrate that the simulation approach can be useful to design storage modules with different types of fibres and arrangements. For example, the preliminary comparison of two different fibres, i.e., carbon and nickel, shows that metal is not always the best choice, as thermal performances are strongly affected by thermal conductivity, volumetric heat capacity, and thermal diffusivity. The storage efficiency of FibC is around 96% compared to the 94% of FibNi for the identical storage cycle. A similar comparison can be made for power density. This is due to the fact that although FibNi and FibC have the same thermal conductivity, in transient heat transfer, the higher heat capacity of FibNi requires more time and more thermal energy for charging. Further development of this approach can be used to study the optimization of fibre arrangement and properties.

From the environmental point of view, the least impacting product was found to be the benchmark PA20 concrete, obtained with an ordinary Portland cement and recycled materials as aggregates. The benchmark plain concrete and the concrete with marble sludge inert material have a similar impact factor, slightly worse than the geopolymer for CO_2_ emission, but generally better in ozone depletion and ecotoxicity. Carbon fibres and Nickel fibres geopolymer matrix composites were found to be the worst, at this stage, due to the high energy demand for fibre production.

On the other hand, these products, with PA0, exhibited the best thermal behaviour from the thermal energy storage point of view, and a careful LCA analysis of the whole TES system, by considering more scenarios and the total lifetime of the system, could suggest ways to reduce the impact factors of the fibre-reinforced geopolymers to exploit their thermal storage benefits, leading to a marked improvement in the environmental performances.

Finally, the main result of this work is that although, theoretically, the addition of long fibres with high thermal properties can improve mechanical resistance and thermal performance, optimizing the charge/discharge times, the combined use of numerical simulation and LCA analysis demonstrates that the addition of fibres can worsen the environmental impact of the overall system, thus reducing the real renewability and sustainability of a storage system.

Thermal and LCA results were used in a multi-criteria decision AHP analysis to simulate the choice of the optimal material. Three different scenarios were simulated, weighing differently the incidence of the environmental impact and the thermal performance. So, depending to the case, the preferred materials are PA20 (showing the lowest environmental impact), PA0 (with a relative intermedia value of environmental impact and very interesting thermal performance), and A4 short-fibred material (the best thermal performing material).

## Figures and Tables

**Table 1:** Thermal properties of storage materials used in simulations.

Material	Density *ρ* (kg/m^3^)	Spec. Heat Cap. *c* (J/kg·K)	Thermal Cond. *k* (W/m·K)	Thermal Diff. *α* × 10^7^ (m^2^/s)	Vol. Thermal Cap. *C_vol_* (kWh/m^3^·K)
**FibC**	1810	800	70	483	1448.24
**FibNi**	8890	456	70	173	4053.84
**G**	1811	751	1.01	7.43	1360.06

**Table 2:** Composition of the investigated materials used in the life cycle assessment (LCA) analysis.

Materials	Unit	C	PA0	PA20	G	FibC	FibNi	A4
**CEM II/A-L 42.5R**	kg/m^3^	280.00	300.00	300.00	-	-	-	-
**Sand**	kg/m^3^	1000.00	-	-	-	-	-	-
**Gravel**	kg/m^3^	400.00	-	-	-	-	-	-
**Fine gravel**	kg/m^3^	200.00	-	-	-	-	-	-
**Marble sludge**	kg/m^3^	-	146.00	171.00	-	-	-	-
**Crushed limestone**	kg/m^3^	-	1648.00	1227.00	1288.83	1234.33	1234.33	-
**Plastic aggregate**	kg/m^3^	-	-	140.00	-	-	-	-
**Fly ash**	kg/m^3^	-	90.00	90.00	313.91	300.63	300.63	-
Alkaline solution *	kg/m^3^	-	-	-	208.27	199.46	199.46	-
Superplasticizer **	L/m^3^	-	6.86	8.91	-	-	-	-
**Fibre**	kg/m^3^	-	-	-	-	88.69	435.61	-
**Calcium aluminate cement**	kg/m^3^	-	-	-	-	-	-	268.00
**Basalt**	kg/m^3^	-	-	-	-	-	-	991.60
**Bauxite**	kg/m^3^	-	-	-	-	-	-	964.80
**Graphite**	kg/m^3^	-	-	-	-	-	-	268.00
**Silica sand**	kg/m^3^	-	-	-	-	-	-	134.00
**Aluminium micropowder**	kg/m^3^	-	-	-	-	-	-	107.20
**Steel**	kg/m^3^	-	-	-	-	-	-	134.00
**Density**	kg/m^3^	2410.00	2190.86	1936.91	1811.00	1823.11	2170.03	2170.03

* Sodium silicate R = 2 (molar SiO_2_/Na_2_O ratio) was used as the alkaline solution. ** Superplasticizer is a mix of Lignosulphonate: max. 35%; Naphthalene sulphonate: max. 30%; Melamine sulphonate: max. 45%; Polycarboxylate: max. 35%. Commercial material from the European Federation of Concrete Admixtures Associations Ltd. (EFCA) (EPD EFCA).

**Table 3:** (**a**) Weights of the sub-criteria variables: A1—climate change, A2—ozone depletion, A3—photochemical oxidant formation, A4—particulate matter, A5—terrestrial ecotoxicity, A6—fossil depletion; (**b**) A7—storage efficiency, A8—ΔT_eff_.

**a**	**A1**	**A2**	**A3**	**A4**	**A5**	**A6**
**A1**	A	3	4	4	5	2
**A2**	1/3	1	2	2	3	1/2
**A3**	1/4	1/2	1	1	2	1/3
**A4**	1/4	1/2	1	1	2	1/3
**A5**	1/5	1/3	1/2	1/2	1	1/4
**A6**	1/2	2	3	3	4	1
**b**	**A7**			**A8**		
**A7**	1			3		
**A8**	1/3			1		

**Table 4:** Thermal performance of storage elements.

Material	ΔT_eff_ (K)	Thermal Storage Efficiency (%)	Volume Power Density (kWh/m^3^)
**FibC**	37.71	95.86	14.24
**FibNi**	36.62	94.63	13.83
**G**	37.11	92.42	13.28

## Data Availability

All data is contained within the article and referred database and/or sources.

## Appendix A

Appendix A is a supplementary section in which all the impacts related to singular species in the analysed materials are shown.

**Table A1:** Plain Cement Concrete (C).

Item	Unit	Total	Concrete C	Cement, CEM II/A-L 42.5R	Gravel, Crushed	Sand	Gravel, Round
Climate change	kg CO_2_ eq	351.17	0.00	323.24	4.83	16.16	6.94
Ozone depletion	kg CFC-11 eq	7.82 × 10^−6^	0.00	6.20 × 10^−6^	2.62 × 10^−7^	9.93 × 10^−7^	3.65 ×10^−7^
Terrestrial acidification	kg SO_2_ eq	1.02	0.00	0.84	0.03	0.10	0.05
Freshwater eutrophication	kg P eq	3.18 × 10^−2^	0.00	2.65 × 10^−2^	1.28 × 10^−3^	3.03 × 10^−3^	9.92 × 10^−4^
Marine eutrophication	kg N eq	4.34 × 10^−2^	0.00	3.53 × 10^−2^	1.30 × 10^−3^	4.83 × 10^−3^	1.97 × 10^−3^
Human toxicity	kg 1,4-DB eq	37.90	0.00	31.26	1.42	3.82	1.41
Photochemical oxidant formation	kg NMVOC	1.05	0.00	0.84	0.03	0.13	0.05
Particulate matter formation	kg PM10 eq	0.40	0.00	0.32	0.01	0.04	0.02
Terrestrial ecotoxicity	kg 1,4-DB eq	9.99 × 10^−3^	0.00	7.75 × 10^−3^	3.98 × 10^−4^	1.35 × 10^−3^	5.01 × 10^−4^
Freshwater ecotoxicity	kg 1,4-DB eq	0.83	0.00	0.62	0.05	0.12	0.04
Marine ecotoxicity	kg 1,4-DB eq	0.90	0.00	0.69	0.04	0.13	0.05
Ionising radiation	kBq U235 eq	18.46	0.00	15.49	0.66	1.79	0.51
Agricultural land occupation	m^2^a	2.45	0.00	1.91	0.11	0.31	0.13
Urban land occupation	m^2^a	3.27	0.00	1.46	0.24	1.12	0.45
Natural land transformation	m^2^	0.04	0.00	0.02	0.00	0.01	0.00
Water depletion	m^3^	417.59	0.12	368.09	11.94	27.02	10.42
Metal depletion	kg Fe eq	6.55	0.00	4.07	0.49	1.42	0.57
Fossil depletion	kg oil eq	42.84	0.00	34.23	1.41	5.12	2.08

**Table A2:** Cement concrete with limestone waste recovery (PA0).

Item	Unit	Total	Concrete PA0	Cement, CEM II/A-L 42.5R	Limestone, Crushed, Washed	Fly Ash	Limestone Residue	Superplasticizer *
Climate change	kg CO_2_ eq	408.63	0.00	377.28	19.20	0.06	−0.83	12.93
Ozone depletion	kg CFC-11 eq	6.80 × 10^−6^	0.00	5.66 × 10^−6^	1.22× 10^−6^	6.76 × 10^−9^	−9.66 × 10^−8^	4.56 × 10^−9^
Terrestrial acidification	kg SO_2_ eq	1.25	0.00	1.09	0.17	0.00	−0.01	0.00
Freshwater eutrophication	kg P eq	0.03	0.00	3.04 × 10^−2^	2.48 × 10^−3^	9.27 × 10^−6^	−7.04 × 10^−5^	6.25 × 10^−6^
Marine eutrophication	kg N eq	0.05	0.00	4.14 × 10^−2^	8.45 × 10^−3^	7.63 × 10^−6^	−2.92 × 10^−4^	5.09 × 10^−6^
Human toxicity	kg 1,4-DB eq	37.67	0.00	34.23	3.45	0.01	−0.08	0.06
Photochemical oxidant formation	kg NMVOC	1.22	0.00	1.00	2.20 × 10^−1^	0.00	−0.01	0.00
Particulate matter formation	kg PM10 eq	0.58	0.00	0.42	1.60 × 10^−1^	0.00	0.00	4.43 × 10^−5^
Terrestrial ecotoxicity	kg 1,4-DB eq	0.01	0.00	0.01	1.60 × 10^−3^	0.00	0.00	2.54 × 10^−5^
Freshwater ecotoxicity	kg 1,4-DB eq	0.77	0.00	0.67	1.09 × 10^−1^	0.00	0.00	1.65 × 10^−4^
Marine ecotoxicity	kg 1,4-DB eq	0.85	0.00	0.73	0.12	0.00	0.00	2.28 × 10^−4^
Ionising radiation	kBq U235 eq	11.82	0.00	10.25	1.64	0.01	−0.09	0.01
Agricultural land occupation	m^2^a	2.11	0.00	1.90	3.41 × 10^−1^	0.00	−0.14	0.00
Urban land occupation	m^2^a	2.47	0.00	1.70	9.16 × 10^−1^	0.00	−0.15	0.00
Natural land transformation	m^2^	0.04	0.00	0.03	5.41 × 10^−3^	0.00	5.95 × 10^−3^	6.30 × 10^−6^
Water depletion	m^3^	458.14	0.15	412.78	45.28	0.28	−0.58	0.23
Metal depletion	kg Fe eq	5.34	0.00	4.06	1.32	0.00	−0.04	0.00
Fossil depletion	kg oil eq	44.60	0.00	38.84	6.26	0.02	−0.53	0.01

* Superplasticizer impacts were taken from the European Federation of Concrete Admixtures Associations Ltd. (EFCA) EPD^®^.

**Table A3:** Cement concrete with limestone and plastic waste recovery (PA20).

Item	Unit	Total	Concrete PA20	Cement, CEM II/A-L 42.5R	Limestone, Crushed, Washed	Fly Ash	Waste Plastic Mixture	Limestone Residue	Superplasticizer *
Climate change	kg CO_2_ eq	78.41	0.00	377.26	14.20	0.06	−328.91	−0.98	16.78
Ozone depletion	kg CFC-11 eq	4.47 × 10^−6^	0.00	5.66 × 10^−6^	9.07 × 10^−7^	6.76 × 10^−9^	−2.00 × 10^−6^	−1.13 × 10^−7^	5.91 × 10^−9^
Terrestrial acidification	kg SO_2_ eq	1.13	0.00	1.09	0.12	2.04 × 10^−4^	−7.51 × 10^−2^	−6.70 × 10^−3^	1.78 × 10^−4^
Freshwater eutrophication	kg P eq	0.03	0.00	0.03	1.80 × 10^−3^	9.27 × 10^−6^	−2.00 × 10^−3^	−8.25 × 10^−5^	8.11 × 10^−6^
Marine eutrophication	kg N eq	0.03	0.00	0.04	6.27 × 10^−3^	7.63 × 10^−6^	−1.38 × 10^−2^	−3.42 × 10^−4^	6.60 × 10^−6^
Human toxicity	kg 1,4-DB eq	5.41	0.00	34.23	2.53	8.29 × 10^−3^	−31.34	−9.80 × 10^−2^	0.07
Photochemical oxidant formation	kg NMVOC	1.06	0.00	1.00	1.64 × 10^−1^	1.31 × 10^−4^	−0.10	−1.04 × 10^−2^	4.84 × 10^−3^
Particulate matter formation	kg PM10 eq	0.51	0.00	0.42	1.19 × 10^−1^	6.64 × 10^−5^	−2.93 × 10^−2^	−3.06 × 10^−3^	5.75 × 10^−5^
Terrestrial ecotoxicity	kg 1,4-DB eq	0.00	0.00	0.01	1.18 × 10^−3^	7.64 × 10^−6^	−6.12 × 10^−3^	−4.53 × 10^−5^	3.30 × 10^−5^
Freshwater ecotoxicity	kg 1,4-DB eq	−4.84	0.00	0.67	8.02 × 10^−2^	2.30 × 10^−4^	−5.58	−2.94 × 10^−3^	2.14 × 10^−4^
Marine ecotoxicity	kg 1,4-DB eq	−3.91	0.00	0.73	8.98 × 10^−2^	2.25 × 10^−4^	−4.73	−3.73 × 10^−3^	2.96 × 10^−4^
Ionising radiation	kBq U235 eq	10.46	0.00	10.25	1.20	0.01	−0.90	−0.11	0.01
Agricultural land occupation	m^2^a	1.83	0.00	1.90	0.25	0.00	−0.16	−0.16	0.00
Urban land occupation	m^2^a	2.13	0.00	1.70	0.68	0.00	−0.08	−0.18	0.00
Natural land transformation	m^2^	0.04	0.00	2.61 × 10^−2^	4.01 × 10^−3^	9.45 × 10^−6^	−6.58 × 10^−4^	6.97 × 10^−3^	8.17 × 10^−6^
Water depletion	m^3^	420.59	0.15	412.76	27.45	0.28	−19.67	−0.68	0.30
Metal depletion	kg Fe eq	4.43	0.00	4.06	0.98	0.00	−0.56	−0.04	0.00
Fossil depletion	kg oil eq	41.41	0.00	38.84	4.63	0.02	−1.48	−0.62	0.01

* Superplasticizer impacts were taken from European Federation of Concrete Admixtures Associations Ltd. (EFCA) EPD^®®^ [46].

**Table A4:** Geopolymer concrete (G).

Item	Unit	Total	Limestone, Crushed, Washed	Fly Ash	Sodium Silicate, without Water, in 37% Solution State	Electricity, Medium Voltage
Climate change	kg CO_2_ eq	269.29	15.02	0.19	239.47	14.61
Ozone depletion	kg CFC-11 eq	1.36 × 10^−5^	9.58 × 10^−7^	2.36 × 10^−8^	1.08 × 10^−5^	1.79 × 10^−6^
Terrestrial acidification	kg SO_2_ eq	1.57	0.13	7.12 × 10^−4^	1.39	5.39 × 10^−2^
Freshwater eutrophication	kg P eq	0.09	1.94 × 10^−3^	3.24 × 10^−5^	8.65 × 10^−2^	2.46 × 10^−3^
Marine eutrophication	kg N eq	0.07	6.62 × 10^−3^	2.66 × 10^−5^	5.71 × 10^−2^	2.00 × 10^−3^
Human toxicity	kg 1,4-DB eq	141.78	2.70	2.89 × 10^−2^	1.37 × 10^−2^	2.19
Photochemical oxidant formation	kg NMVOC	1.03	0.17	4.58 × 10^−4^	0.82	0.03
Particulate matter formation	kg PM10 eq	0.61	0.13	2.32 × 10^−4^	0.46	0.02
Terrestrial ecotoxicity	kg 1,4-DB eq	0.02	1.25 × 10^−3^	2.67 × 10^−5^	0.02	2.03 × 10^−3^
Freshwater ecotoxicity	kg 1,4-DB eq	4.17	8.50 × 10^−2^	8.03 × 10^−4^	4.02	0.06
Marine ecotoxicity	kg 1,4-DB eq	4.14	9.50 × 10^−2^	7.85 × 10^−4^	3.99	0.06
Ionising radiation	kBq U235 eq	19.03	1.28	3.09 × 10^−2^	15.36	2.35
Agricultural land occupation	m^2^a	18.45	0.27	3.27 × 10^−3^	17.93	0.25
Urban land occupation	m^2^a	4.48	0.72	7.65 × 10^−4^	3.72	0.05
Natural land transformation	m^2^	0.05	4.23 × 10^−3^	3.30 × 10^−5^	0.04	2.48 × 10^−3^
Water depletion	m^3^	441.39	35.43	9.82 × 10^−1^	329.98	74.99
Metal depletion	kg Fe eq	33.23	1.03	5.10 × 10^−3^	31.82	0.38
Fossil depletion	kg oil eq	65.16	4.89	5.95 × 10^−2^	55.70	4.50

**Table A5:** Geopolymer concrete with carbon fibre (FibC).

Item	Unit	Total	Limestone, Crushed, Washed	Fly Ash	Sodium Silicate	Carbon PAN Fibre	Electricity, Medium Voltage (IT)
Climate change	kg CO_2_ eq	2107.91	14.38	0.18	229.29	1849.46	14.60
Ozone depletion	kg CFC-11 eq	2.41 × 10^−4^	9.17 × 10^−7^	2.26 × 10^−8^	1.03 × 10^−5^	2.28 × 10^−4^	1.79 × 10^−6^
Terrestrial acidification	kg SO_2_ eq	8.32	0.12	6.82 × 10^−4^	1.33	6.81	0.05
Freshwater eutrophication	kg P eq	0.39	1.86 × 10^−3^	3.10 × 10^−5^	0.08	0.30	2.46 × 10^−3^
Marine eutrophication	kg N eq	0.32	6.33 × 10^−3^	2.55 × 10^−5^	0.05	0.25	2.00 × 10^−3^
Human toxicity	kg 1,4-DB eq	386.96	2.59	0.03	131.04	251.12	2.19
Photochemical oxidant formation	kg NMVOC	5.32	0.17	4.39 × 10^−4^	0.79	4.33	3.41 × 10^−2^
Particulate matter formation	kg PM10 eq	2.79	0.12	2.22 × 10^−4^	0.44	2.21	1.74 × 10^−2^
Terrestrial ecotoxicity	kg 1,4-DB eq	0.28	1.20 × 10^−3^	2.55 × 10^−5^	1.84 × 10^−2^	0.26	2.03 × 10^−3^
Freshwater ecotoxicity	kg 1,4-DB eq	11.28	8.14 × 10^−2^	7.69 × 10^−4^	3.85	7.28	6.04 × 10^−2^
Marine ecotoxicity	kg 1,4-DB eq	11.02	9.10 × 10^−2^	7.51 × 10^−4^	3.82	7.05	5.90 × 10^−2^
Ionising radiation	kBq U235 eq	317.69	1.23	2.96 × 10^−2^	1.47 × 10^1^	299.37	2.35
Agricultural land occupation	m^2^a	48.77	0.26	3.13 × 10^−3^	1.72 × 10^1^	31.09	0.25
Urban land occupation	m^2^a	10.15	0.69	7.32 × 10^−4^	3.56	5.86	4.64 × 10^−2^
Natural land transformation	m^2^	0.36	4.05 × 10^−3^	3.16 × 10^−5^	3.95 × 10^−2^	0.32	2.48 × 10^−3^
Water depletion	m^3^	9954.70	33.92	0.94	3.16 × 10^2^	9528.94	74.95
Metal depletion	kg Fe eq	73.97	0.99	4.88 × 10^−3^	3.05 × 10^1^	42.13	0.38
Fossil depletion	kg oil eq	635.34	4.69	0.06	5.33 × 10^1^	572.76	4.50

**Table A6:** Geopolymer concrete with Nickel fibre (FibNi).

Item	Unit	Total	Limestone, Crushed, Washed	Fly Ash	Sodium Silicate, without Water, in 37% Solution State	Fe-Ni-Cr Alloy	Electricity Medium Voltage
Climate change	kg CO_2_ eq	2.64 × 10^3^	14.38	0.18	229.29	2371.01	14.60
Ozone depletion	kg CFC-11 eq	1.18 × 10^−4^	9.17 × 10^−7^	2.26 × 10^−8^	1.03 × 10^−5^	1.03 × 10^−4^	1.79 × 10^−6^
Terrestrial acidification	kg SO_2_ eq	4.32 × 10^2^	0.12	6.82 × 10^−4^	1.33	430.63	0.05
Freshwater eutrophication	kg P eq	5.20	1.86 × 10^−3^	3.10 × 10^−5^	0.08	5.11	2.46 × 10^−3^
Marine eutrophication	kg N eq	1.50	6.33 × 10^−3^	2.55 × 10^−5^	0.05	1.43	2.00 × 10^−3^
Human toxicity	kg 1,4-DB eq	9.04 × 10^3^	2.59	0.03	131.04	8899.00	2.19
Photochemical oxidant formation	kg NMVOC	5.02 × 10^1^	0.17	4.39 × 10^−4^	0.79	49.22	0.03
Particulate matter formation	kg PM10 eq	9.82 × 10^1^	0.12	2.22 × 10^−4^	0.44	97.64	0.02
Terrestrial ecotoxicity	kg 1,4-DB eq	1.50	1.20 × 10^−3^	2.55 × 10^−5^	0.02	1.48	2.03 × 10^−3^
Freshwater ecotoxicity	kg 1,4-DB eq	2.86 × 10^2^	8.14 × 10^−2^	7.69 × 10^−4^	3.85	282.33	6.04 × 10^−2^
Marine ecotoxicity	kg 1,4-DB eq	2.93 × 10^2^	9.10 × 10^−2^	7.51 × 10^−4^	3.82	289.46	5.90 × 10^−2^
Ionising radiation	kBq U235 eq	3.18 × 10^2^	1.23	2.96 × 10^−2^	14.71	298.18	2.35
Agricultural land occupation	m^2^a	1.40 × 10^2^	0.26	3.13 × 10^−3^	17.17	122.02	0.25
Urban land occupation	m^2^a	6.44 × 10^1^	0.69	7.32 × 10^−4^	3.56	60.05	0.05
Natural land transformation	m^2^	4.47 × 10^−1^	0.00	3.16 × 10^−5^	0.04	0.40	0.00
Water depletion	m^3^	5.10 × 10^4^	33.92	0.94	315.96	50,494.71	74.95
Metal depletion	kg Fe eq	9.41 × 10^3^	0.99	4.88 × 10^−3^	30.46	9379.32	0.38
Fossil depletion	kg oil eq	6.48 × 10^2^	4.69	5.70 × 10^−2^	53.33	582.52	4.50

**Table A7:** A4 material.

Item	Unit	Total	Basalt	Bauxite	Graphite	Calcium Aluminate Cement *	Silica Sand	Aluminium Micropowder	Steel, Low-Alloyed, Hot Rolled	Electricity Medium Voltage
Climate change	kg CO_2_ eq	2548.18	17.73	152.05	10.83	302.90	3.46	1829.45	188.51	43.24
Ozone depletion	kg CFC-11 eq	0.00	1.10 × 10^−6^	9.84 × 10^−6^	6.06 × 10^−7^	7.18 × 10^−9^	2.41 × 10^−7^	1.54 × 10^−4^	7.44 × 10^−6^	5.33 × 10^−6^
Terrestrial acidification	kg SO_2_ eq	15.17	0.14	1.94	0.08	0.00	0.02	11.97	0.86	0.16
Freshwater eutrophication	kg P eq	1.12	3.42 × 10^−3^	1.48 × 10^−2^	2.69 × 10^−3^	9.84 × 10^−6^	6.28 × 10^−4^	9.76 × 10^−1^	1.11 × 10^−1^	7.04 × 10^−3^
Marine eutrophication	kg N eq	0.81	0.01	0.06	3.10 × 10^−3^	8.02 × 10^−6^	7.12 × 10^−4^	0.69	0.04	5.90 × 10^−3^
Human toxicity	kg 1,4-DB eq	1182.36	3.56	16.77	2.66	0.37	0.74	981.76	170.62	5.87
Photochemical oxidant formation	kg NMVOC	9.46	0.17	1.79	0.07	0.11	0.02	6.29	0.90	0.10
Particulate matter formation	kg PM10 eq	6.79	0.10	0.75	0.04	6.98 × 10^−5^	0.01	5.16	0.68	0.05
Terrestrial ecotoxicity	kg 1,4-DB eq	0.13	0.00	1.13 × 10^−2^	0.00	1.52 × 10^−4^	0.00	0.08	2.05 × 10^−3^	5.96 × 10^−3^
Freshwater ecotoxicity	kg 1,4-DB eq	53.26	0.11	0.54	0.07	3.24 × 10^−4^	0.02	46.29	6.05	0.17
Marine ecotoxicity	kg 1,4-DB eq	49.78	0.12	0.81	0.08	7.94 × 10^−4^	0.02	42.63	5.96	0.16
Ionising radiation	kBq U235 eq	176.59	2.35	13.95	1.54	9.41 × 10^−3^	0.29	138.51	12.95	7.00
Agricultural land occupation	m^2^a	48.19	0.26	1.50	0.28	9.88 × 10^−4^	0.13	41.44	3.86	0.73
Urban land occupation	m^2^a	47.68	8.41	4.47	0.33	1.86 × 10^−4^	0.11	31.30	2.93	0.14
Natural land transformation	m^2^	0.83	0.48	0.03	0.00	9.92 × 10^−6^	0.00	0.29	0.02	7.37 × 10^−3^
Water depletion	m^3^	15,296.41	33.07	139.41	23.36	0.90	3.06	13,820.80	1053.02	222.79
Metal depletion	kg Fe eq	356.72	0.87	29.13	0.66	1.53 × 10^−3^	0.20	73.91	250.97	0.99
Fossil depletion	kg oil eq	533.72	5.60	50.18	3.18	0.02	0.95	417.10	43.30	13.39

* Calcium aluminate cement impacts were taken from çimsa RESISTO40^®®^ Calcium Aluminate Cement EPD^®^ [45].

## Appendix B

Appendix B is the section in which all the criteria ratios for Super-decision analysis are shown.

**Table A8:** Climate change criteria.

	PA20	PA0	C	A4	G	FibC	FibNi
PA20	1	3	4	8	2	7	9
PA0	1/3	1	2	5	1/3	4	6
C	1/4	1/2	1	4	1/2	4	5
A4	1/8	1/5	1/4	1	1/7	½	2
G	1/2	3	2	7	1	5	8
FibC	1/9	1/6	1/6	2	1/5	1	3
FibNi	1/7	1/4	1/4	1/2	1/8	1/3	1

Inconsistency 0.0227.

**Table A9:** Ozone depletion criteria.

	PA20	PA0	C	A4	G	FibC	FibNi
PA20	1	1	1/2	6	3	7	7
PA0	1	1	1/3	6	3	7	7
C	3	3	1	8	2	9	7
A4	1/6	1/6	1/8	1	1/7	2	1/2
G	1/3	1/3	1/2	7	1	8	2
FibC	1/7	1/7	1/9	1/2	1/8	1	1/2
FibNi	1/7	1/5	1/7	2	1/3	2	1

Inconsistency 0.0298.

**Table A10:** Particulate matter criteria.

	PA20	PA0	C	A4	G	FibC	FibNi
PA20	1	1	1/3	4	1/3	3	7
PA0	1	1	1/3	4	1/3	3	6
C	3	3	1	6	1	3	9
A4	1/4	1/4	1/6	1	1/6	1	2
G	2	3	1	6	1	3	9
FibC	1/3	1/3	1/9	1	1/9	1	2
FibNi	1/7	1/6	1/7	1/2	1/7	1/2	1

Inconsistency 0.00775.

**Table A11:** Photochemical oxidant formation criteria.

	PA20	PA0	C	A4	G	FibC	FibNi
PA20	1	1	1/2	3	1/2	2	7
PA0	1	1	1/2	3	1/2	2	7
C	2	2	1	4	1	3	9
A4	1/3	1/3	1/4	1	1/4	1/2	2
G	2	2	1	4	1	3	9
FibC	1/2	1/2	1/3	2	1/2	1	5
FibNi	1/7	1/7	1/9	1/2	1/9	1/5	1

Inconsistency 0.0106.

**Table A12:** Marine ecotoxicity criteria.

	PA20	PA0	C	A4	G	FibC	FibNi
PA20	1	2	1/2	7	1/2	4	9
PA0	1/2	1	1/2	5	1/2	3	7
C	1/2	1	1	5	1	3	7
A4	1/7	1/5	1/4	1	1/4	1/2	2
G	1/3	1/2	1	4	1	2	5
FibC	1/4	1/3	1/3	2	1/3	1	2
FibNi	1/7	1/7	1/9	1/2	1/9	1/5	1

Inconsistency 0.0158.

**Table A13:** Fossil depletion criteria.

	PA20	PA0	C	A4	G	FibC	FibNi
PA20	1	2	2	7	3	9	9
PA0	1/2	1	1	6	2	7	7
C	1/2	1	1	6	2	7	7
A4	1/7	1/6	1/6	1	1/4	2	2
G	1/3	1/2	1/2	4	1	5	5
FibC	1/9	1/7	1/7	1/2	1/7	1	1
FibNi	1/9	1/7	1/7	1	1/7	1	1

Inconsistency 0.0165.

**Table A14:** Storage efficiency criteria.

	PA20	PA0	C	A4	G	FibC	FibNi
PA20	1	1/4	3	1/6	1/2	1/4	1/3
PA0	4	1	8	1/2	3	1	2
C	1/3	1/8	1	1/9	1/4	1/8	1/6
A4	6	2	9	1	4	2	3
G	2	1/3	4	1/4	1	1/3	1/2
FibC	4	1	8	1/2	3	1	2
FibNi	3	1/2	6	1/3	2	1/2	1

Inconsistency 0.01567.

**Table A15:** ΔT_eff_.

	PA20	PA0	C	A4	G	FibC	FibNi
PA20	1	1/4	2	1/4	1/2	1/3	1
PA0	4	1	7	1	3	2	4
C	1/2	1/7	1	1/7	1/4	1/5	1/2
A4	4	1	7	1	3	2	4
G	2	1/3	4	1/3	1	1/2	2
FibC	3	1/2	5	1/2	2	1	3
FibNi	1	1/4	2	1/4	1/2	1/3	1

Inconsistency 0.0035.

**Table A16:** LCA criteria.

	*Climate Change*	Ozone Depletion	Particulate Matter	Photochemical Oxidant Formation	Marine Ecotoxicity	Fossil Depletion
*Climate change*	1	3	4	4	5	2
ozone depletion	1/3	1	2	2	3	1/2
particulate matter	1/4	1/2	1	1	2	1/3
Photochemical oxidant formation	1/4	1/2	1	1	2	1/3
Marine ecotoxicity	1/5	1/3	1/2	1/2	1	¼
Fossil depletion	1/2	2	3	3	4	1

Inconsistency 0.01151.

**Table A17:** Thermal performance criteria.

	*Storage Efficiency*	ΔT_eff_
*Storage efficiency*	1	3
ΔT_eff_	1/3	1

**Table A18:** Local normalized priority.

	*Climate Change*	Ozone Depletion	Particulate Matter	Photochemical Oxidant Formation	Marine Ecotoxicity	Fossil Depletion	Storage Efficiency	ΔT_eff_
*A4*	0.04	0.03	0.04	0.06	0.03	0.04	0.32	0.29
C	0.16	0.35	0.28	0.26	0.21	0.31	0.02	0.03
FibC	0.05	0.02	0.06	0.09	0.07	0.03	0.20	0.17
FibNi	0.03	0.04	0.02	0.02	0.03	0.03	0.12	0.06
G	0.24	0.24	0.28	0.26	0.13	0.20	0.08	0.10
PA20	0.36	0.15	0.16	0.15	0.33	0.20	0.05	0.06
PA0	0.13	0.15	0.16	0.15	0.21	0.20	0.20	0.30

**Table A19:** Global results for the three different scenarios from Super-decisions analysis.

**LCA: Thermal Performance 50:50**			
**Name**	**Ideals**	**Normals**	**Raw**
**A4**	0.87	0.17	0.06
**C**	0.70	0.14	0.05
**FibC**	0.63	0.12	0.04
**FibNi**	0.38	0.07	0.02
**G**	0.82	0.16	0.05
**PA20**	0.79	0.15	0.05
**PA0**	1.00	0.19	0.06
**LCA: Thermal Performance 80:20**			
**Name**	**Ideals**	**Normals**	**Raw**
**A4**	0.42	0.09	0.03
**C**	0.93	0.20	0.07
**FibC**	0.36	0.08	0.03
**FibNi**	0.22	0.05	0.02
**G**	0.93	0.20	0.07
**PA20**	1.00	0.21	0.07
**PA0**	0.82	0.17	0.06
**LCA: Thermal Performance 20:80**			
**Name**	**Ideals**	**Normals**	**Raw**
**A4**	1.00	0.24	0.08
**C**	0.28	0.07	0.02
**FibC**	0.68	0.17	0.06
**FibNi**	0.41	0.10	0.03
**G**	0.47	0.11	0.04
**PA20**	0.37	0.09	0.03
**PA0**	0.87	0.21	0.07
